# Supplementary material for: Grain-growth mediated hydrogen sorption kinetics and compensation effect in single Pd nanoparticles
Source: Nat Commun. 2021 Sep 14;12:5427. doi: 10.1038/s41467-021-25660-x (PMC8440611; doi:10.1038/s41467-021-25660-x)
Supplement: Supplementary file 1 — Supplementary Information [file 41467_2021_25660_MOESM1_ESM.pdf]

Supplementary Information

Grain-Growth Mediated Hydrogen Sorption Kinetics and Compensation Effect in Single Pd Nanoparticles

Svetlana Alekseeva[1], Michal Strach[1], Sara Nilsson[1], Joachim Fritzsche[1], Vladimir P. Zhdanov[1,2], Christoph Langhammer[1,*]

[1]Department of Physics, Chalmers University of Technology, 412 96 Göteborg, Sweden
[2]Boreskov Institute of Catalysis, Russian Academy of Sciences, Novosibirsk 630090, Russia

*Correspondence to: clangham@chalmers.se

Table of contents

1. Grazing Incidence X-ray Diffraction (GIXRD) of as-deposited Pd disks............................3

2. Vacuum setup schematics..3

3. Extraction procedure for t_{50}..4

4. Measurement scheme for mixed T-sweep..4

5. t_{50} vs. T plots fitted with NLLS..5

6. Arrhenius plots fitted with LLS..5

7. Arrhenius parameters extracted with LLS and NLLS methods..7

8. Goodness-of-fit statistics for LLS and NLLS methods..7

9. Distribution of plateau pressures at absorption and desorption..8

10. Average t_{50} values as function of measurement number at each T............................10

11. Figure 5 analog for absorption..11

12. CQF analysis..12

13. Correlation of a kinetics slowing factor with E_a..17

14. Data set size and CQF values..18

References..19

1. Grazing Incidence X-ray Diffraction (GIXRD) of as-deposited Pd disks

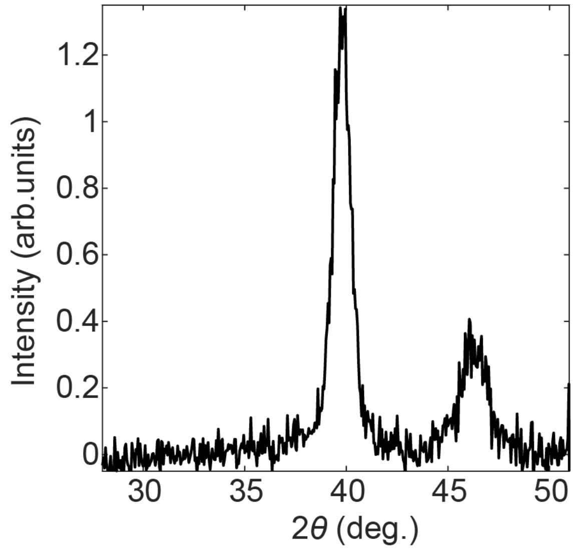

Supplementary Figure 1. XRD pattern of as-deposited Pd nanodisks, which reveals average crystallite size of 10 ± 2 nm.

2. Vacuum setup schematics

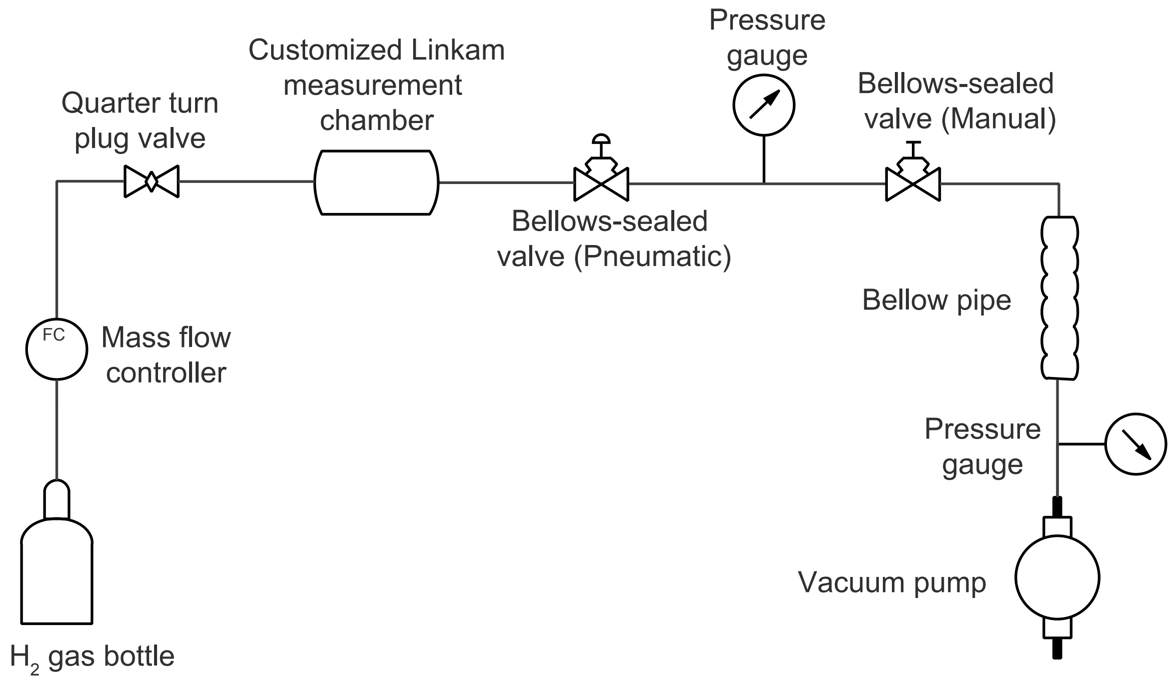

Supplementary Figure 2. Schematics of the vacuum setup used in the experiments. A customized Linkam temperature-controlled vacuum chamber was positioned on a motorized stage on the upright optical microscope.

3. Extraction procedure for t_{50}

In order to extract t_{50} of the signal we used a function for mid-reference level crossing for bilevel waveform (Supplementary Figure 3).

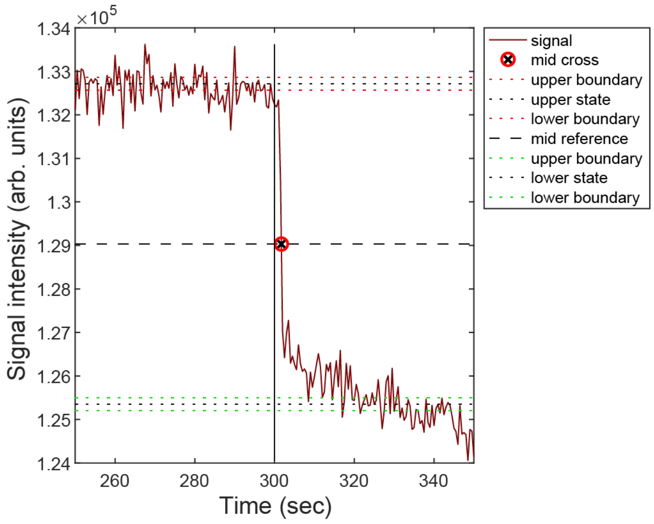

Supplementary Figure 3. Example of a H_2 absorption trace, where the Matlab “midcross” function is used to extract t_{50} (cross outlined with red circle). The vertical line indicates time stamp for introduction of H_2.

4. Measurement scheme for mixed *T*-sweep

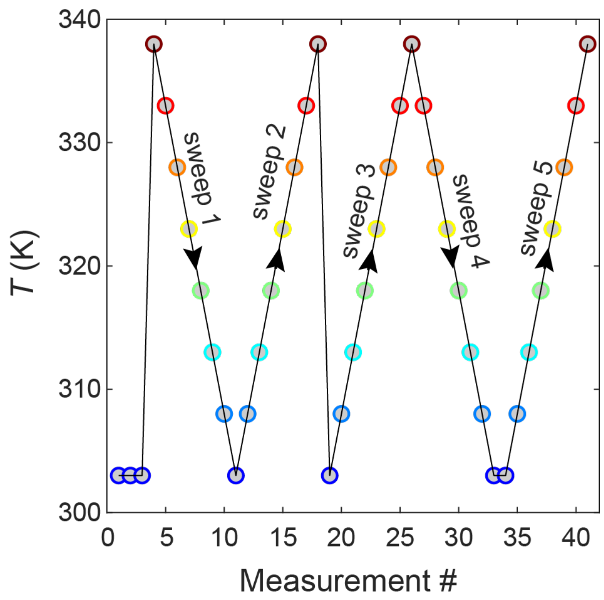

Supplementary Figure 4. Order of measurements of t_{50} both for H_2 absorption and desorption in mixed *T*-sweep samples. The results of the first three subsequent measurement points at 303 K are shown in Figure 1 in the main text.

5. t_{50} vs. T plots fitted with NLLS

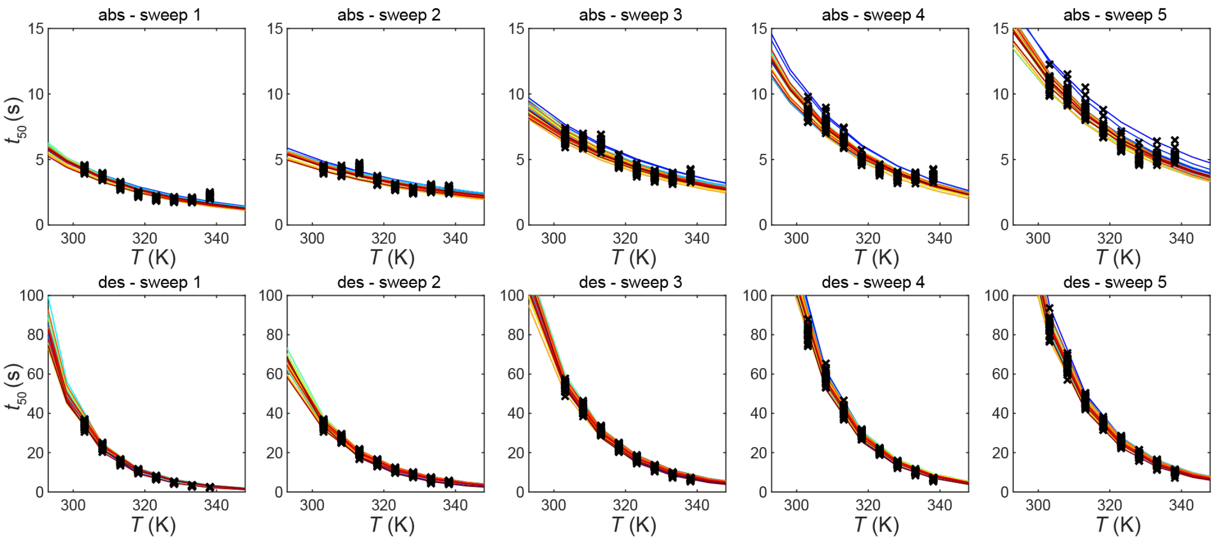

Supplementary Figure 5. t_{50} vs. T plot (crosses) for 24 single Pd particles from 1st to 5th T-sweep (left to right, with mixed T-sweep according to Supplementary Figure 4) fitted with non-linear least squares regression (NLLS) for each individual particle (colored lines). The upper and lower panels correspond to absorption and desorption respectively.

6. Arrhenius plots fitted with LLS

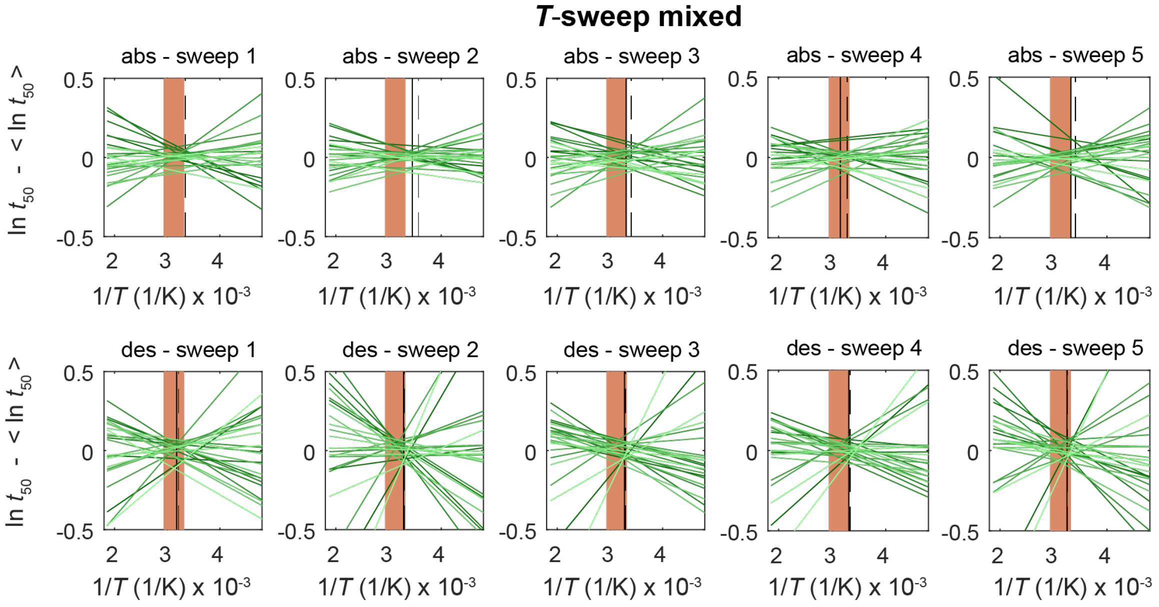

Supplementary Figure 6. Arrhenius plots for 24 particles (shades of green lines) from 1st to 5th T-sweep (left to right, with T-sweep direction according to Supplementary Figure 4) constructed from Arrhenius parameters by fitting least squares linear regression to experimental values of ln t_{50} versus the inverse temperature. For clarity at each temperature the average < ln t_{50} > taken over all measured 24 particles is subtracted from the individual ln t_{50}. The upper and lower panels correspond to absorption and desorption data respectively. The brown rectangle

indicates the experimental T range. Vertical lines indicate T_{min} (solid) and T_{isokin} (dashed) according to analysis by Griessen et al.[1]

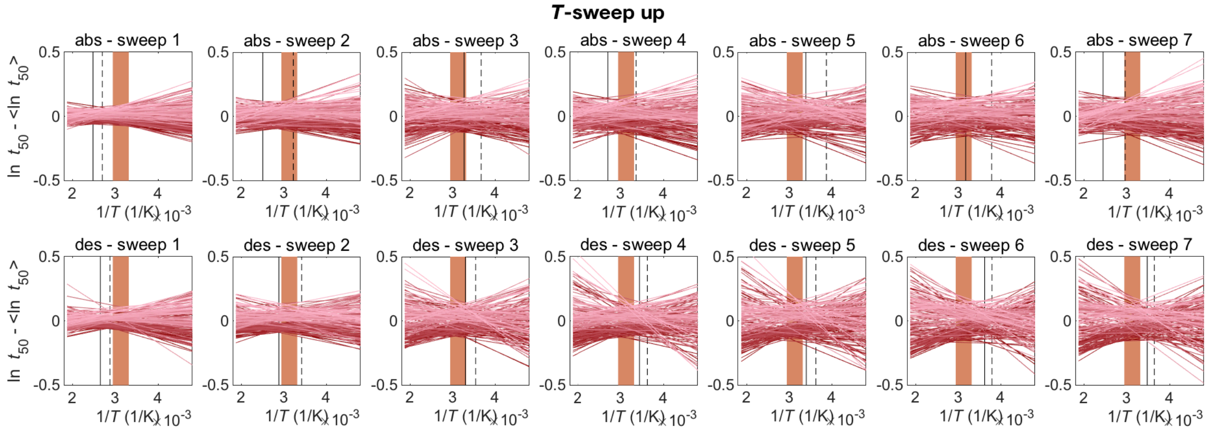

Supplementary Figure 7. Arrhenius plots for 180 particles (shades of red lines) from 1st to 7th T-sweep up (left to right) constructed from Arrhenius parameters by fitting least squares linear regression to experimental values of ln t_{50} versus the inverse temperature. For clarity at each temperature the average < ln t_{50} > taken over all measured 180 particles is subtracted from the individual ln t_{50}. The upper and lower panels correspond to absorption and desorption data respectively. The brown rectangle indicates the experimental T range. Vertical lines indicate T_{min} (solid) and T_{isokin} (dashed) according to analysis by Griessen et al.[1]

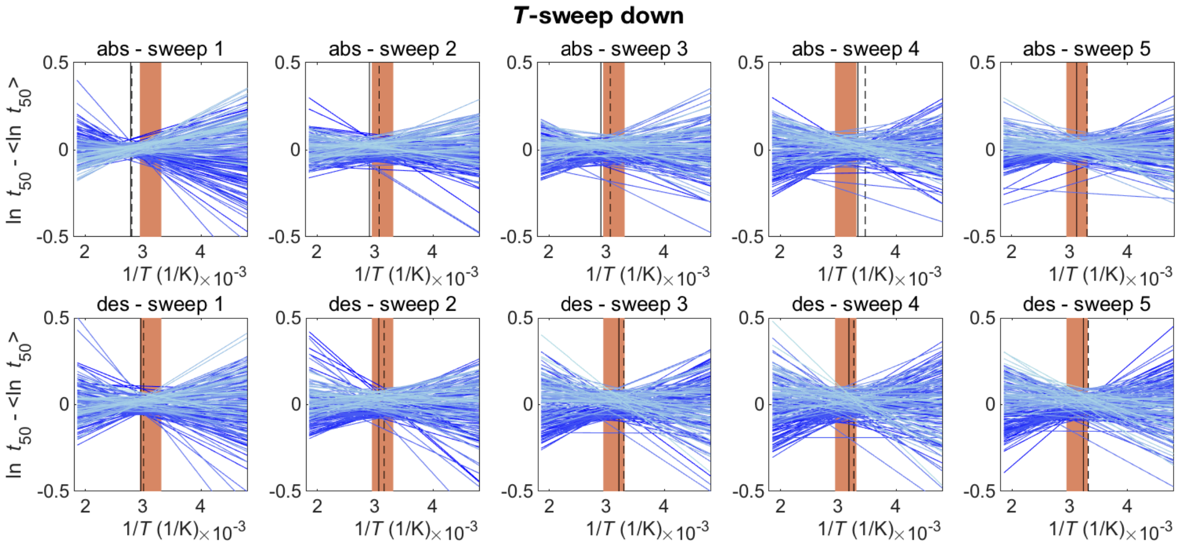

Supplementary Figure 8. Arrhenius plots for 180 particles (shades of blue lines) from 1st to 5th T-sweep down (left to right) constructed from Arrhenius parameters by fitting least squares linear regression to experimental values of ln t_{50} versus the inverse temperature. For clarity at each temperature the average < ln t_{50} > taken over all measured 180 particles is subtracted from the individual ln t_{50}. The upper and lower panels correspond to absorption and desorption data respectively. The brown rectangle indicates the experimental T range. Vertical lines indicate T_{min} (solid) and T_{isokin} (dashed) according to analysis by Griessen et al.[1]

7. Arrhenius parameters extracted with LLS and NLLS methods

Data were analysed with both linear (LLS) and nonlinear least squares regression (NLLS) methods in order to see whether there are significant differences between the results[2]. In our case, both methods result in similar trends (Supplementary Figure 9), however the goodness-of-fit statistics are better for the LLS method (Supplementary Figure 10).

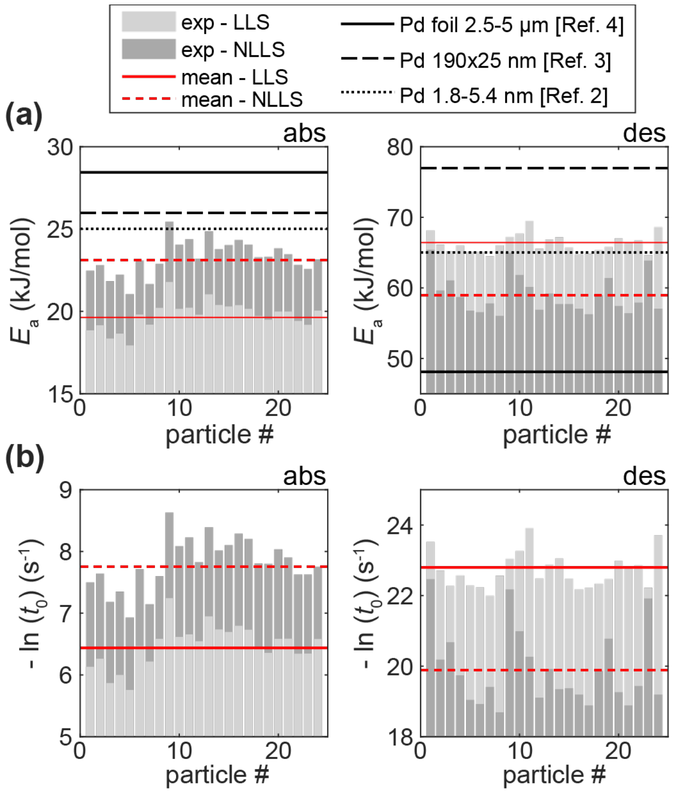

Supplementary Figure 9. (a) Apparent activation energy, E_a, and (b) pre-exponential factor, t_0, at absorption (left) and desorption (right) extracted for the 24 individual Pd disks (light grey and dark grey bars are data from LLS and NLLS fits respectively with their mean value indicated as red solid and dashed line). The data were extracted from the 1st *T*-sweep as shown in Supplementary Figure 4, which included a full set of consecutive measurements across the T range of the experiment from 338 to 303 K. Black lines in the upper panel denote E_a values for annealed Pd nanoparticles[3] of 2-5 nm in size (dotted), annealed Pd disks[4] of 190 nm in diameter and 25 nm in height (dashed) and Pd foil[5] of 2.5-5 μm in thickness (solid). The corresponding Arrhenius plots and nonlinear fits to data can be found in Supplementary Figures 5 & 6. See goodness-of-fit statistics for both methods in Supplementary Figure 10.

8. Goodness-of-fit statistics for LLS and NLLS methods

The goodness-of-fit of a model describes how well it fits the set of observations. The following are the plots with goodness-of-fit statistics for each *T*-sweep, which include sum of square errors (SSE), R-square, adjusted R-square and Root Mean Squared Error (RMSE). These values were extracted using the Matlab Curve Fitting Toolbox™ software.

SSE measures the total deviation of the response values from their fit. A value closer to 0 indicates that the model has a smaller random error component, and that the fit will be more useful for prediction.
R-square measures how successful the fit is in explaining the variation of the data, or in other words, it is the square of the correlation between the response values and the predicted response values. R-square can take on any value between 0 and 1, with a value closer to 1 indicating that a greater proportion of variance is accounted for by the model.
The adjusted R-square uses the R-square statistic defined above, and adjusts it based on the residual degrees of freedom. The residual degrees of freedom is defined as the number of response values *n* minus the number of fitted coefficients *m* estimated from the response values ($v = rv - fc$). *v* indicates the number of independent pieces of information involving the *rv* data points that are required to calculate the sum of squares. The adjusted R-square statistic can take on any value less than or equal to 1, with a value closer to 1 indicating a better fit.
RMSE is also known as the fit standard error and the standard error of the regression. It is an estimate of the standard deviation of the random component in the data, and just as with *SSE*, a mean square error value closer to 0 indicates a fit that is more useful for prediction.

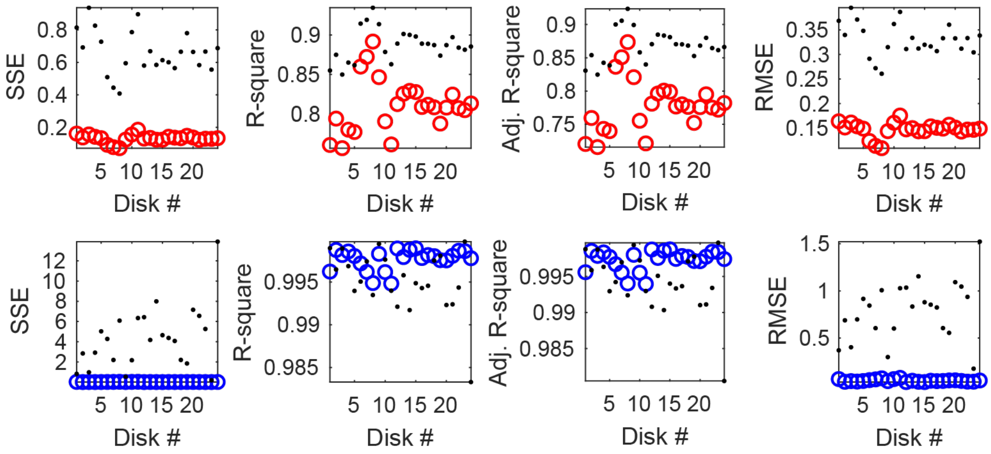

Supplementary Figure 10. Goodness-of-fit statistics for the 1st *T*-sweep as shown in Supplementary Figure 4. The statistics are presented for both methods that were used to extract Arrhenius parameters, i.e., least square linear regression (LLS – red circles for absorption, blue circles for desorption) and nonlinear least square regression (NLLS – black dots).

9. Distribution of plateau pressures at absorption and desorption

In addition to the kinetic measurements, it is instructive to assess the evolution of the thermodynamics of hydrogen absorption and desorption in our samples. Following this line, we have measured sorption isotherms at 303 K (Supplementary Figure 11) for a sample comprised of an array of 24 single Pd nanodisks of the same size as in kinetic measurements, which before the measurement were not exposed to any (de)hydrogenation cycles. The obtained data were then compared with a corresponding isotherm measurement on a sample that had been cycled 41 times in kinetic measurements prior to the isotherm measurement (i.e., sample T_{mix} after

kinetics measurements). We use separate samples for this purpose, i.e., we cannot use as-deposited sample that underwent isotherm measurement to cycle it in kinetics measurements – the kinetics results of such sample will be different, since exposure to H_2 during an isotherm measurement of an as-deposited sample will inevitably change it, and therefore it cannot be used for comparison with a sample that was deposited with Pd and then cycled directly (without prior isotherm measurement). The samples are then measured not in vacuum, but in a gas flow mode (i.e., at atmospheric pressure), with step-wise increase/decrease in hydrogen partial pressure, and at each pressure step there is dwelling time in order to allow the particles reach stable state at this pressure step. From the isotherm measurements we extracted the plateau pressures for absorption and desorption (P_{abs} and P_{des}) for each particle and observe sizable increase in hysteresis for the cycled sample, as well as a larger spread in P_{abs} and P_{des} values for the individual particles. This distinct increase in hysteresis further supports the idea of grain growth in the particles upon cycling[6].

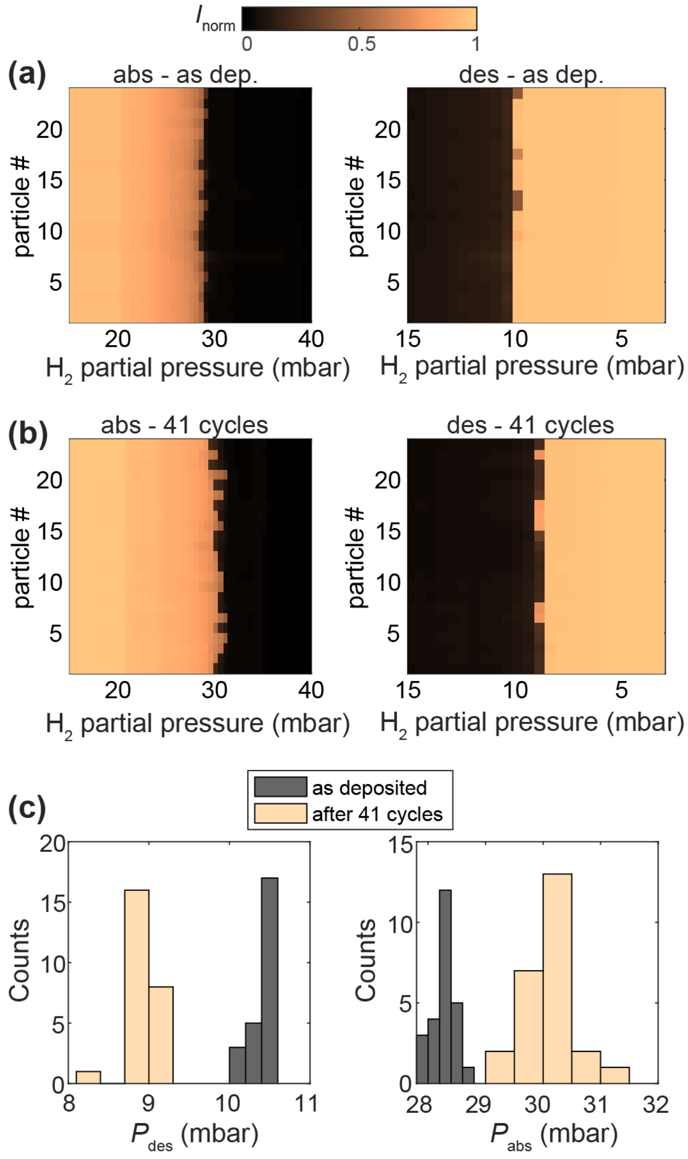

Supplementary Figure 11. Individual normalized intensity (I_{norm}) and hydrogen partial pressure isotherms at absorption (left) and desorption (right) for (a) as-deposited sample and (b) sample

cycled 41 times prior to isotherm measurements. (c) Distribution of (left) desorption and (right) absorption plateau pressures measured at 303 K on a sample in as-deposited condition (dark-grey) and after 41 (de)hydrogenation cycles (light-orange). Cycling with hydrogen clearly increases the hysteresis and the spread in plateau pressure values, which confirms grain growth.

10. Average t_{50} values as function of measurement number at each T

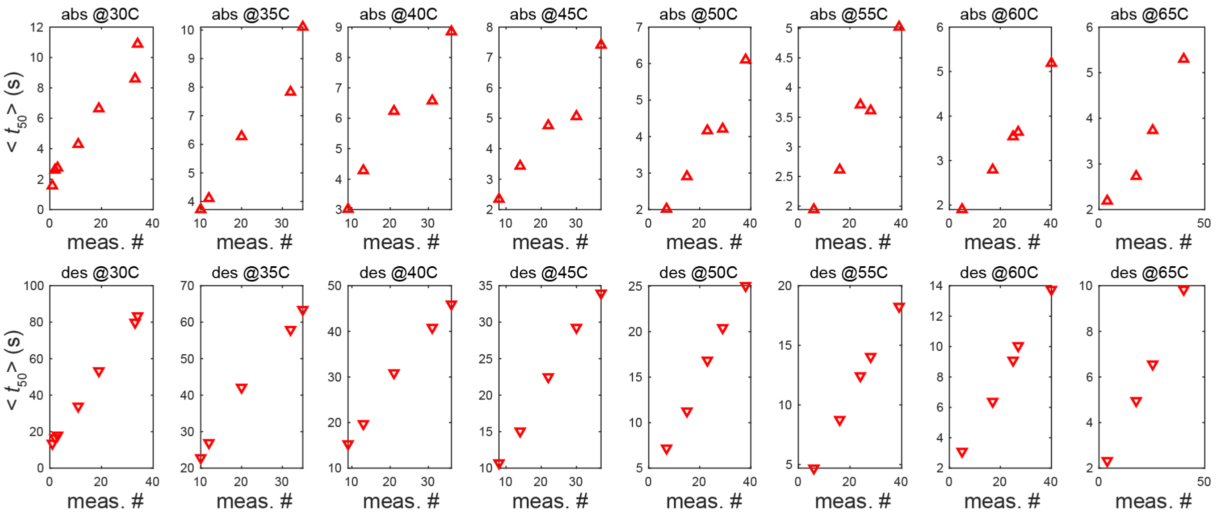

Supplementary Figure 12. Average t_{50} values become higher with each measurement for all measured temperatures and both for absorption (top row) and desorption (bottom row). Measurement numbers are given according to the experiment scheme depicted in Supplementary Figure 4.

11. Figure 5 analog for absorption

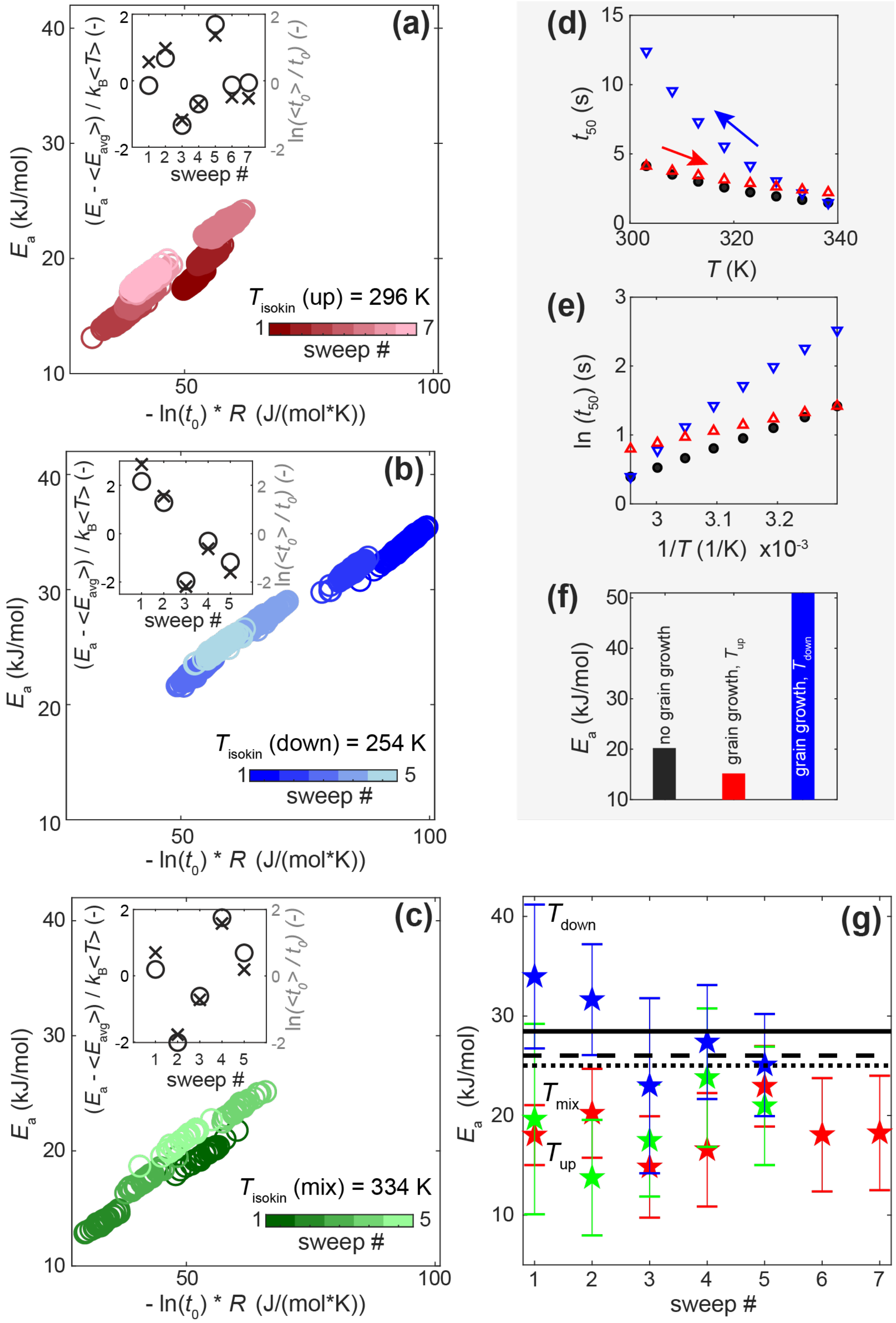

Supplementary Figure 13. Modified Cremer-Constable plots at absorption for three samples measured with different T-sweeps: (a) up – red; (b) down – blue and (c) mixed – green. Each subsequent T-sweep in a set is colored as a lighter shade of the main color (colorbar). Insets show normalized change in activation energy, $E_a - \langle E_{avg}\rangle / k_B T$ (where E_a is the average value for 180 (T_{up} and T_{down}) or 24 particles (T_{mix}) in each sweep, $\langle E_{avg}\rangle$ is the average of the entire

set and k_B is the Boltzmann constant, black stars), and logarithm of the pre-exponential factor ($\ln(<t_0>/t_0)$, where t_0 is the average value for the particles in each sweep and $<t_0>$ is the average of the entire set, gray stars) as function of measured T-sweeps. T_{isokin} for all data measured with each of the T-sweep directions (T_{mix}, T_{down} and T_{up}) lumped together was calculated according to Ref.[1] In (d-f) we simulate 3 scenarios for derivation of the activation energy at desorption: ideal case without grain growth (black circles), grain growth with T-sweep up (red triangles) and grain growth with T-sweep down (blue triangles): (d) t_{50} vs. T, (e) Arrhenius plots of $\ln(t_{50})$ vs. $1/T$ and (f) corresponding activation energy for each scenario. (g) Average E_a of the particles for each of the measured sweep directions (colored stars). Black lines denote E_a values at absorption for annealed Pd nanoparticles[3] of 2-5 nm in size (dotted), annealed Pd disks[4] of 190 nm in diameter and 25 nm in height (dashed) and Pd foil[5] of 2.5-5 µm in thickness (solid). (e) Compensation Quality Factor (CQF) for each set of the T-sweep directions in relation to threshold level γ at 95, 99 and 99.5 % confidence levels (dotted, dashed and solid lines, respectively), which shows that it is significantly high for the T-sweep down set, indicating non-statistical origin of the compensation effect according to Ref. 1.

12. CQF analysis

This section describes the analysis developed by Griessen et al.[1] (Eq. 28-35 therein), which we applied to characterize the compensation effect observed between Arrhenius parameters in our data. The analysis requires calculation of parameters such as Compensation Quality Factor (CQF), which depends on the number of samples in the measurement (in our case, number of particles measured in one T-sweep ($N = 24$ or 180), coefficient of determination value (R_{square}), as well as variance in and covariance between Arrhenius parameters.

Analytically calculated variance in $\ln(t_{50})$ for the set of the N investigated particles allows to determine the temperature T_{min} at which the variance of $\ln(t_{50})$ reaches a minimum. The variance of $\ln(t_{50})$ at T_{min} is a direct measure of the degree of coalescence of the Arrhenius plots. The ratio of the variance of $\ln(t_{50})$ at T_{min} normalized to the largest experimentally measured $\ln(t_{50})$ variance defines a CQF that characterizes quantitatively the extent of the crossing region of Arrhenius lines. The CQF is by definition unity for perfect compensation ($T_{min} = T_{isokin}$, where T_{isokin} is the isokinetic temperature that corresponds to the slope of the Constable plot (i.e., the temperature at which all the particles in the specific measurement set have the same rate of reaction[1]) and tends towards zero when the Arrhenius lines do not come close to a single crossing. The calculated value of CQF is also compared to a threshold value γ, which depends on N and the choice of the confidence level (i.e., level of certainty, %).

According to this analysis, if CQF $< \gamma$ at the chosen confidence level, an observed compensation effect is a statistical artefact. This is the case for our absorption and desorption data if we analyse each T-sweep *separately* at a 99.5% confidence level (see Supplementary Figures 6-8 where there is no well-defined crossing of Arrhenius plots for each of the independent T-sweeps). Accordingly, when we apply this analysis to the data presented in Fig. 2 in the main text, i.e., the 1st decreasing T-sweep for 24 single particles, CQF is lower than γ (at 99.5 % confidence level) in both cases. This implies that if analysed for this specific data set, the observed compensation effect is of statistical origin, despite Cremer-Constable plots

showing high level of correlation, with relatively high $R_{\text{square}} \geq 0.98$, both for absorption and desorption data (Supplementary Figure 14). However, as we discuss in the main text by invoking a larger set of particles to increase N in this analysis (180 vs. 24), in fact, the compensation effect can be traced back to particle-specific grain structure prior to the very first hydrogenation and therefore has non-statistical origin (SI Section 13).

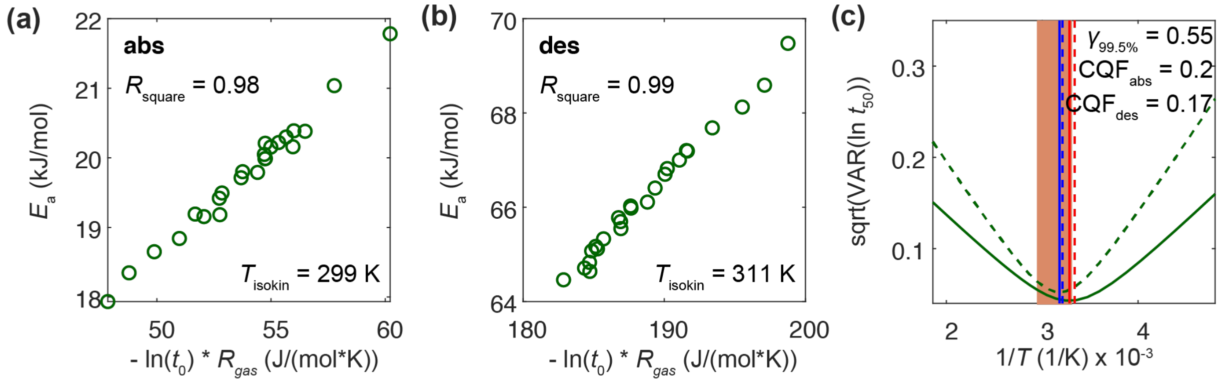

Supplementary Figure 14. Modified Cremer-Constable plots at (a) absorption and (b) desorption for the data presented in Fig. 2 in the main text (1st sweep with decreasing temperature in T-sweep mixed dataset). (c) Temperature dependence of the spread of ln(t_{50}) values calculated according to Griessen et al.[1] (green lines, solid – absorption, dashed - desorption). Vertical lines indicate T_{isokin} (dashed) and T_{min} (solid) for absorption (red) and desorption (blue). T_{min} indicates where the minimum spread of ln(t_{50}) values occurs. The brown rectangle indicates T_{exp} (303-338 K), where the spread of ln(t_{50}) is little, and which leads to low CQF values of ~0.2, both for absorption and desorption, indicating statistical compensation effect.

Similarly, we have also applied this CQF analysis to the scenario where we include whole series of T-sweeps with specific T-sweep directions (i.e., not just a single T-sweep) to maximize the grain growth effect (see stars in Supplementary Figures 15-17 d, e). Also, this analysis then reveals a non-statistical origin of the compensation effect for desorption with 99.5% confidence level for "T-sweeps up and down", as well as for absorption with "T-sweep down", thereby identifying grain growth as the physical mechanism behind the observed compensation effect. We attribute the failure of the analysis to identify the compensation effect as non-statistical for the case of absorption for "T-sweep up" to the larger experimental error in absorption measurements, since the absorption process tends to be much faster than desorption, which leads to less accurate t_{50} data. This is especially pronounced for measurements at higher T, where we are close to the time the resolution limit of our instrument. For the mixed T-sweep case, the CQF is low also for desorption because the Arrhenius parameters tend to oscillate between different T-sweep directions, which also implies a higher ratio between minimum and maximum of t_{50} variance, and therefore by definition leads to a low CQF value.

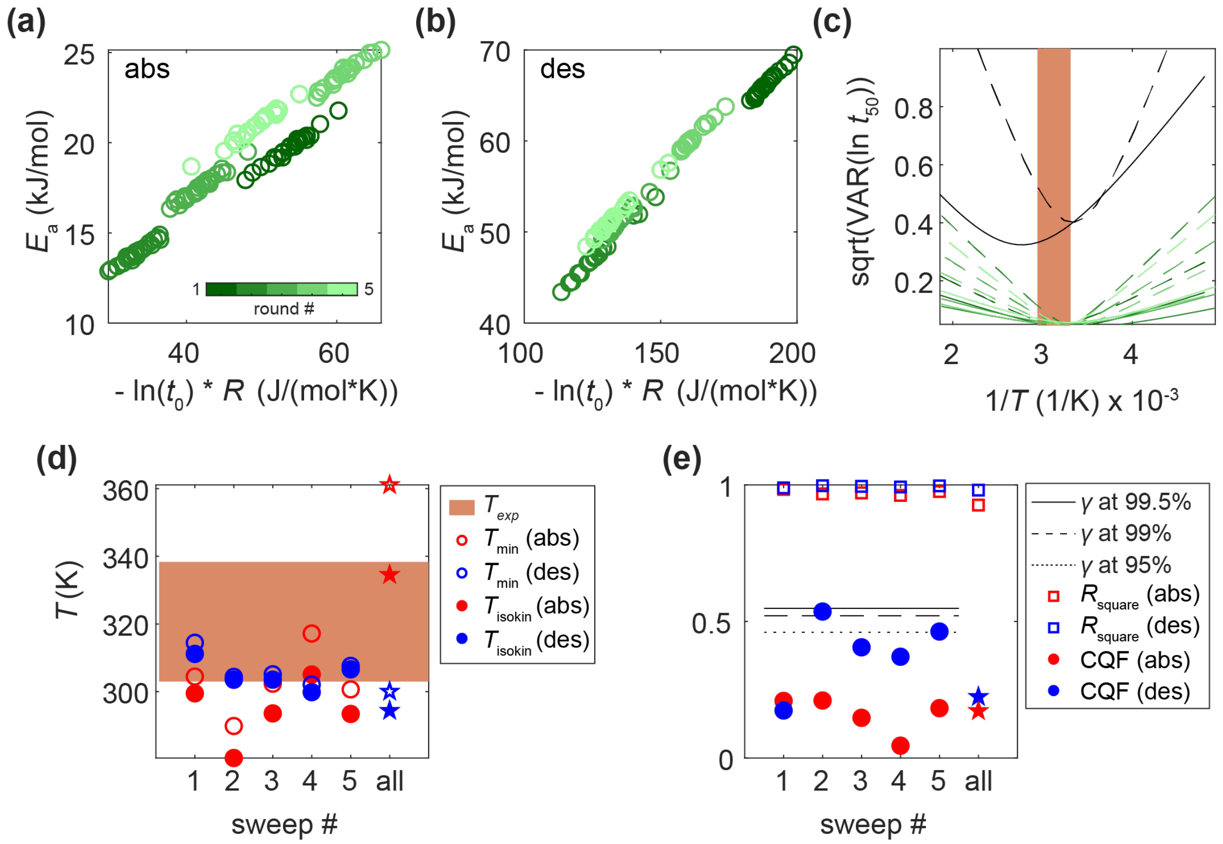

Supplementary Figure 15. Modified Cremer-Constable plot of the Arrhenius parameters (color bars) for 24 measured particles from 1st to 5th T-sweeps at (a) absorption and (b) desorption using mixed T-sweep (according to Supplementary Figure 4). (c) Temperature dependence of the spread of ln(t_{50}) values calculated according Ref.[1] for each of the 5 sweeps (color code is the same as in (a,b)). The spread for all sweeps together is shown in black lines (solid and dashed lines indicate abs/des data respectively). (d) The slope of the modified Cremer-Constable plots is called isokinetic temperature T_{isokin} (full circles). The minimum spread of ln(t_{50}) values occurs at T_{min} (hollow circles). Brown rectangle in (c) and (d) indicates the temperature range of the experiment from 303 to 338 K (T_{exp}). (e) Within this range the spread of ln(t_{50}) values is small for each sweep separately, which leads to low CQF values (full circles) that are below the threshold value γ at confidence levels of 95% (dotted line), 99% (dashed line) and 99.5 % (solid line) for each separate sweep and for both absorption and desorption. R_{square} for each sweep is indicated as squares. Red and blue markers indicate absorption and desorption data respectively. The stars in (d, e) indicate values calculated for the entire set of 5 T-sweeps lumped together. The CQF values for the entire set are low due to lower variance in the data caused by the mixed directions of the individual T-sweeps in the set.

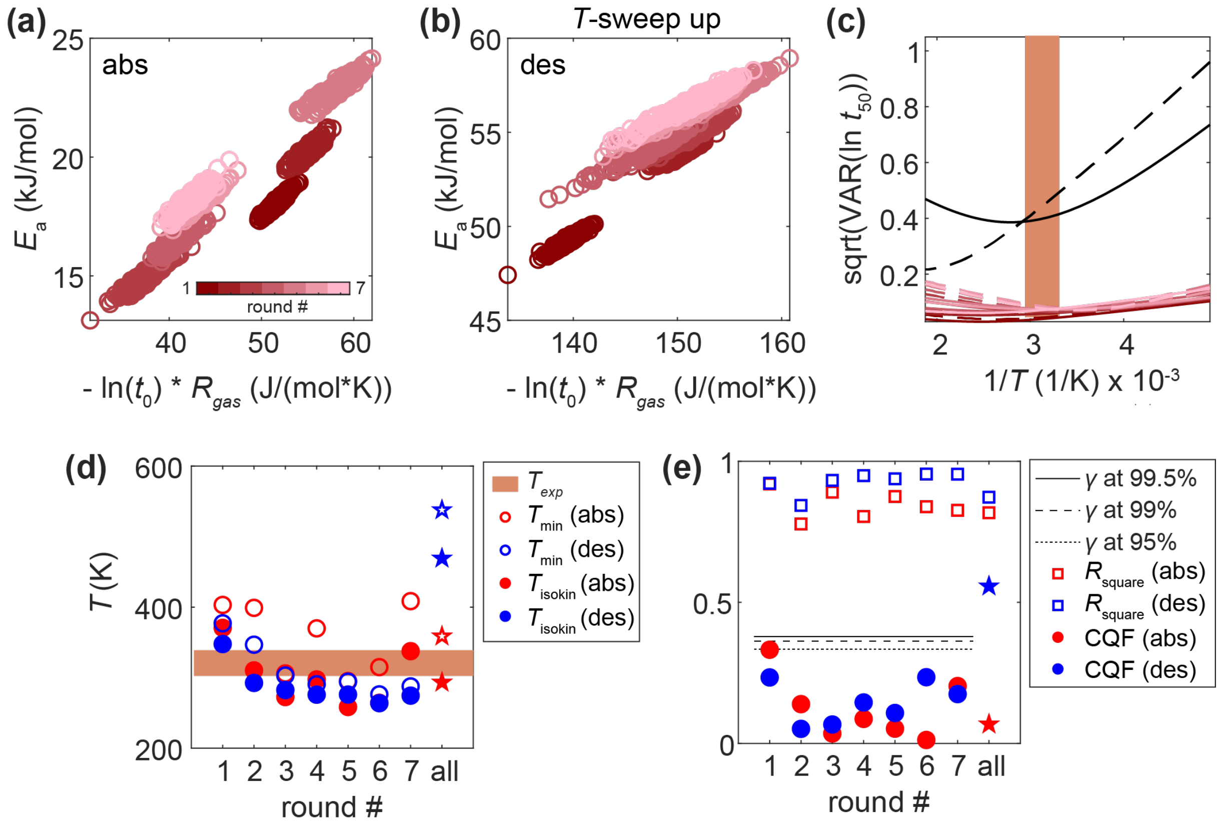

Supplementary Figure 16. Modified Cremer-Constable plot of the Arrhenius parameters (color bars) for 180 measured single particles from 1st to 7th T-sweep at (a) absorption and (b) desorption using T-sweep up. (c) Temperature dependence of the spread of ln(t_{50}) values calculated according to Ref.[1] for each of the 7 sweeps (color code is the same as in (a,b)). The spread for all sweeps together is shown in black lines (solid and dashed lines indicate abs/des data respectively). (d) The slope of the modified Cremer-Constable plots is called isokinetic temperature T_{isokin} (full circles). The minimum spread of ln(t_{50}) values occurs at T_{min} (hollow circles). Brown rectangle in (c) and (d) indicates the temperature range of the experiment from 303 to 338 K (T_{exp}). (e) Within this range the spread of ln(t_{50}) values is small for each sweep separately, which leads to low CQF values (full circles) that are below the threshold value γ at confidence levels of 95% (dotted line), 99% (dashed line) and 99.5 % (solid line) for each separate sweep and for both absorption and desorption. R_{square} for each sweep is indicated as squares. Red and blue markers indicate absorption and desorption data, respectively. The stars in (d, e) indicate values calculated for entire set of 7 T-sweeps lumped together. The CQF values for the entire set are high for desorption and low for absorption, which we attribute to higher experimental error in absorption measurements of t_{50} in general.

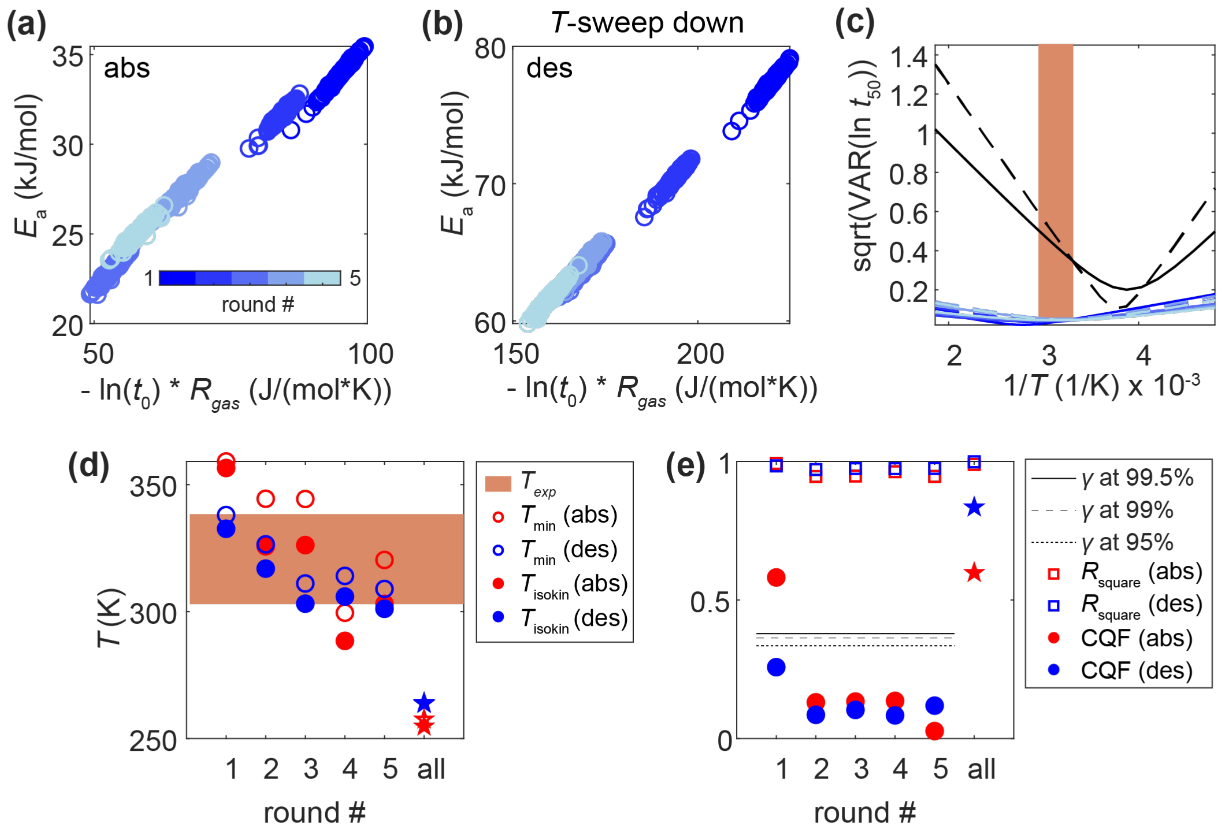

Supplementary Figure 17. Modified Cremer-Constable plot of the Arrhenius parameters (color bars) for 180 measured single particles from 1st to 5th T-sweeps at (a) absorption and (b) desorption using T-sweep down. (c) Temperature dependence of the spread of ln(t_{50}) values calculated according to Ref.[1] for each of the 5 sweeps (color code is the same as in (a,b)). The spread for all sweeps together is shown in black lines (solid and dashed lines indicate abs/des data respectively). (d) The slope of the modified Cremer-Constable plots is called isokinetic temperature T_{isokin} (full circles). The minimum spread of ln t_{50} values occurs at T_{min} (hollow circles). Brown rectangle in (c) and (d) indicates the temperature range of the experiment 303 to 338 K (T_{exp}). (e) Within this range the spread of ln(t_{50}) values is small for each sweep separately, which leads to low CQF values (full circles) that are below the threshold value γ at confidence levels of 95% (dotted line), 99% (dashed line) and 99.5 % (solid line) for each separate sweep and for both absorption and desorption. R_{square} for each sweep is indicated as squares. Red and blue markers indicate absorption and desorption data, respectively. The stars in (d, e) indicate values calculated for the entire set of 5 T-sweeps lumped together. The CQF values for the entire set are high both for absorption and desorption.

13. Correlation of a kinetics slowing factor with E_a

The slowing factor (SF) is defined as the ratio of the latest t_{50} measured at 303 K to the first t_{50} measured at 303 K. All three samples used for sweeps T_{up}, T_{down} and T_{mix} were pre-cycled 3 times with H_2 at 303 K before sets of T-sweeps for extraction of Arrhenius parameters were performed. Similar to Fig. 6 in the main text, where SF versus E_a values at the first T-sweep is plotted, in Supplementary Figure 18 we plot SF vs. E_a for all the other corresponding sweeps not shown in the main text.

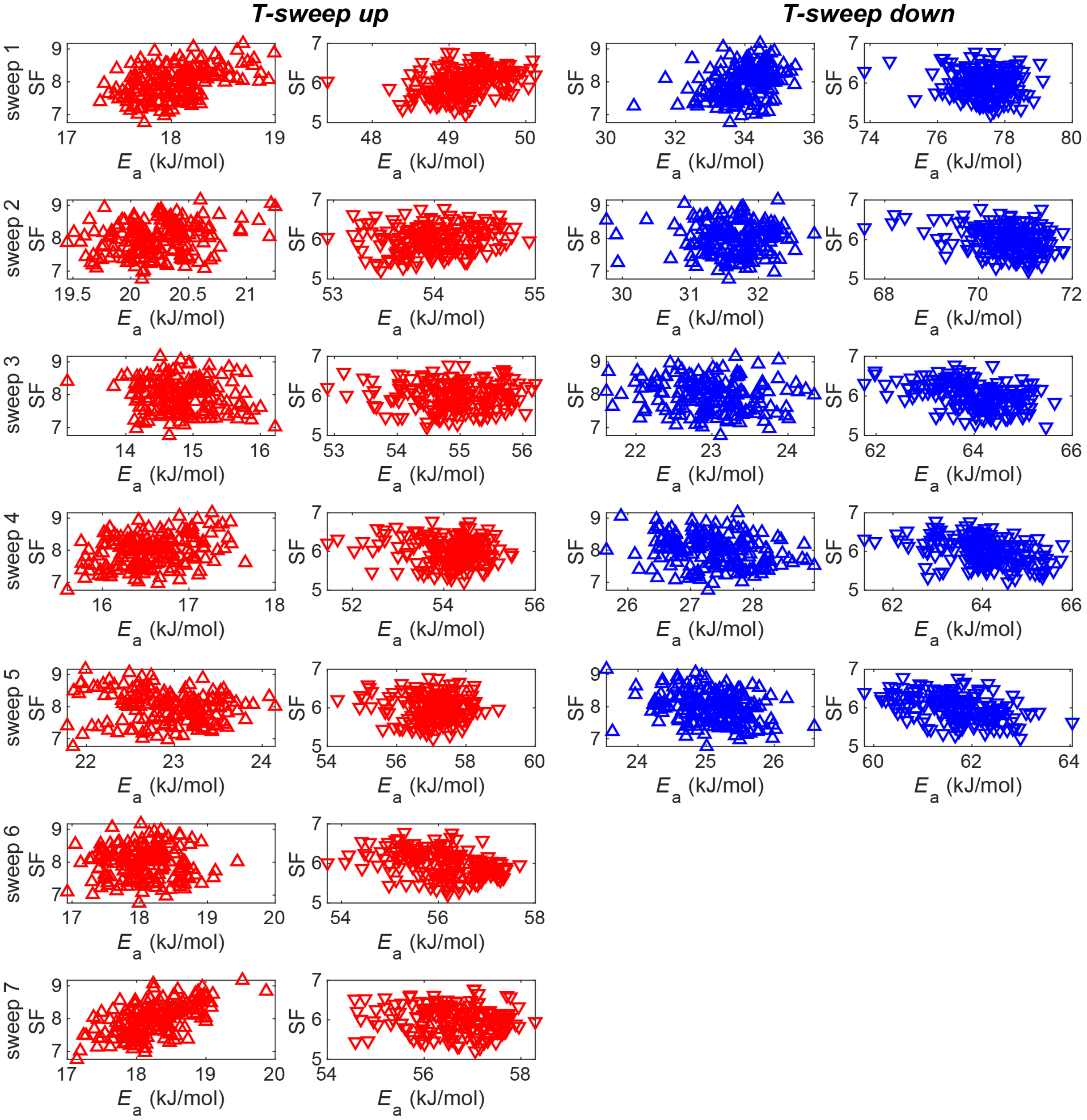

Supplementary Figure 18. Slowing factor (t_{50} (last) / t_{50} (first) at 303 K) versus activation energy E_a at the 1st sweep for samples measured according to scheme T_{up} (left panel, red data) and T_{down} (right panel, blue data) at absorption (upward pointing triangles) and desorption (downward pointing triangles). For T_{up} SF = t_{50} (53)/ t_{50} (1) and T_{down} SF = t_{50} (43)/ t_{50} (1), where numbers in parentheses indicate the cycle number at which the corresponding last and first measurement at 303 K was performed for each sample.

14. Data set size and CQF values

To illustrate the influence of sample size on the calculated CQF value within a single T-sweep, we use the dataset with 180 particles measured using a T_{down} sweep, where the correlation between slowing factor and activation energy was the most pronounced (Fig. 6b in the main text). For this purpose, we randomly divide the dataset of 180 particles into 7 subsets each consisting of 25 particles, where the 1st set includes particles 1 to 25, the 2d set – particles 26 to 50 and so on, while the 7th set includes particles 150 to 175. Particle numbers indicate their position on the sample. Then we plot corresponding SF vs. E_a (Supplementary Figure 19a, b) for each of the particle subsets. We see that with fewer particles included in the analysis it becomes difficult to see the correlation between the two parameters. We also calculate the CQF value for each of the 7 subsets and compare it to CQF value of the entire set of 180 particles (Supplementary Figure 19c). The comparison shows that depending on the constituent nanoparticles in the subset, the CQF value can be greatly different than the value of the entire 180-particle set both for absorption and desorption, highlighting the importance of large data sets (N-value) if this analysis is to be reliably applied.

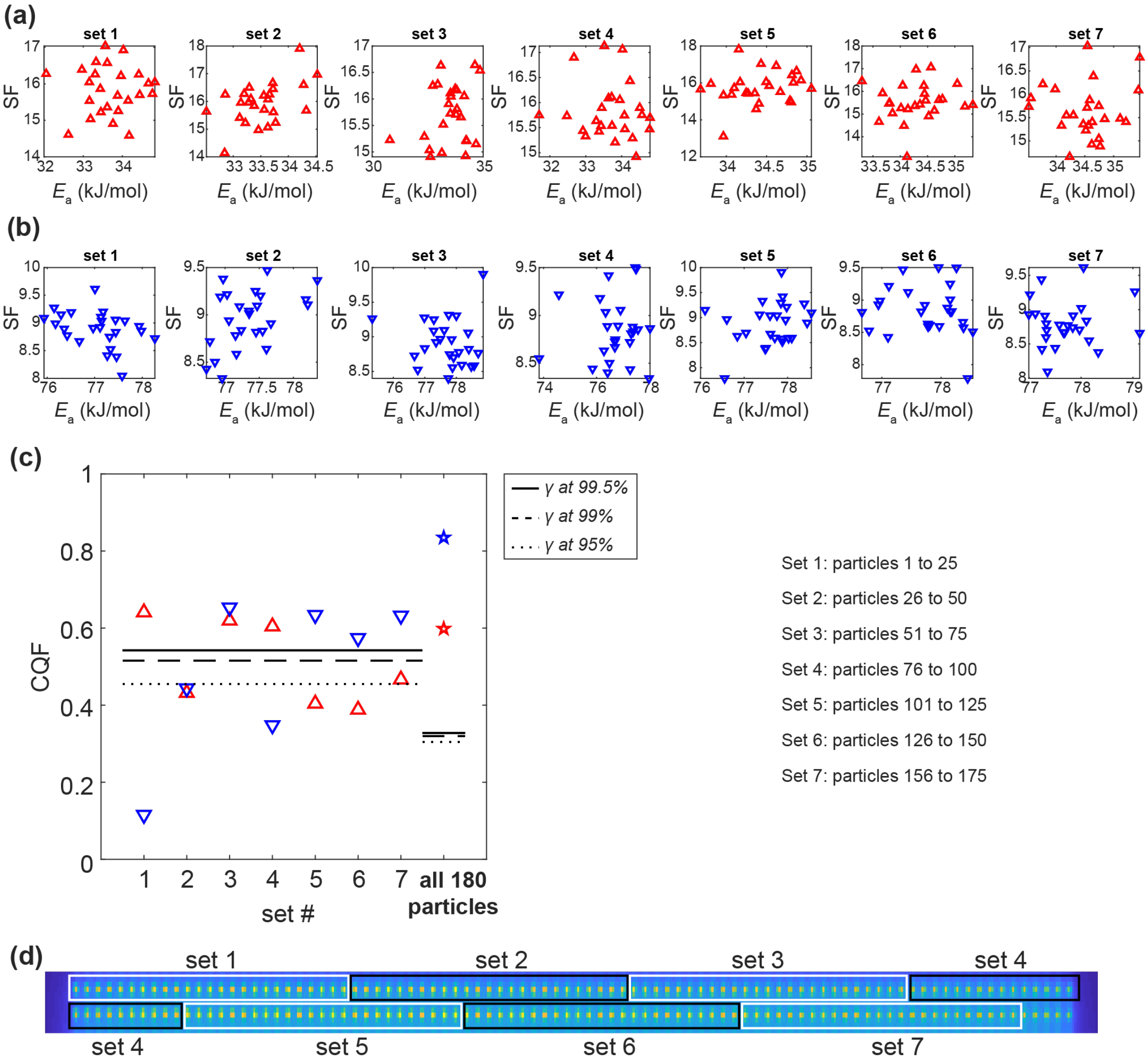

Supplementary Figure 19. Slowing factor, SF = (t_{50} (43) / t_{50} (1) at 303 K vs. E_a, obtained from the 1st T-sweep for a sample of 180 particles measured according to T_{down} scheme, divided into 7 sets of 25 particles, at (a) absorption and (b) desorption. Numbers in parentheses for SF indicate the cycle number at which the corresponding last and first measurement at 303 K was performed for each sample. (c) CQF value calculated for each of the 7 subsets (triangles) and for the entire 180-particle set (stars) in relation to the threshold level γ at 95, 99 and 99.5 % confidence levels (dotted, dashed and solid lines, respectively and according to number of particles in the set, i.e., 25 and 180) according to Ref. 1 (d) CCD image of the sample with 180 particles with black and white boxes indicating corresponding particle subsets.

References

1 Griessen, R. *et al.* Single Quality Factor for Enthalpy-Entropy Compensation, Isoequilibrium and Isokinetic Relationships. *ChemPhysChem* **21**, 1618-1618, (2020).

2 Barrie, P. J. The mathematical origins of the kinetic compensation effect: 1. the effect of random experimental errors. *Physical Chemistry Chemical Physics* **14**, 318-326, (2012).

3 Langhammer, C., Zhdanov, V. P., Zorić, I. & Kasemo, B. Size-Dependent Kinetics of Hydriding and Dehydriding of Pd Nanoparticles. *Physical Review Letters* **104**, 135502 (2010).

4 Nugroho, F. A. A. *et al.* Metal–polymer hybrid nanomaterials for plasmonic ultrafast hydrogen detection. *Nature Materials* **18**, 489-495, (2019).

5 Auer, W. & Grabke, H. J. The Kinetics of Hydrogen Absorption in Palladium (α- and β-phase) and Palladium-Silver-Alloys. *Berichte der Bunsengesellschaft für physikalische Chemie* **78**, 58-67, (1974).

6 Alekseeva, S. *et al.* Grain boundary mediated hydriding phase transformations in individual polycrystalline metal nanoparticles. *Nature Communications* **8**, 1084, (2017).
